# Editorial: Extracellular Matrix Dynamics in Biology, Bioengineering, and Pathology

**DOI:** 10.3389/fcell.2020.00759

**Published:** 2020-08-21

**Authors:** Rajprasad Loganathan, Charles D. Little, Brenda J. Rongish

**Affiliations:** ^1^Department of Cell Biology, Johns Hopkins University, Baltimore, MD, United States; ^2^Department of Anatomy and Cell Biology, University of Kansas Medical Center, Kansas City, KS, United States

**Keywords:** ECM, matrisome, EMT, stem cell, angiogenesis, rheometry, bioscaffold

## Introduction

Research aimed at understanding the nature of extracellular matrix (ECM) dynamics has been, in recent years, a shared endeavor that is spread across many disciplines including biology, bioengineering, and pathology. Novel findings have illuminated our current understanding that the ECM is a dynamic composite; this emerging concept challenges the conventional school of thought that the ECM is a passive/static substrate for cellular functions. These advances have come from the students of ECM adopting an interdisciplinary approach to investigate its role in multiple model systems. The collection of articles published under the Research Topic, “Extracellular matrix dynamics in biology, bioengineering, and pathology,” is an exemplar of the collective effort advanced by investigators bridging the interdisciplinary divides.

## Biology

Bres and Faissner, in their comprehensive review, discuss Low Density Receptor-Related Protein 1 (Lrp1)—a functionally versatile member of the low density lipoprotein receptor family. Its role extends beyond ligand uptake, receptor mediated endocytosis, and endocytosis transport. Indeed, Lrp1 interacts with the ECM with consequential effects for integrin-mediated cell adhesion and tissue plasminogen activator-mediated ECM dynamics in the nervous system.

Huss et al. present their compilation of the quail matrisome genes, which we expect will serve as a standard reference guide for the ECM research community. It will be of significant utility for investigating the roles of ECM dynamics in avian embryogenesis, for which quail embryos have served as a critical model system. Using a multidisciplinary approach, Huss et al., also report that the avian primordial germ cell motion occurs in the backdrop of a dynamic (motile) ECM fibril meshwork—its architecture dependent on integrin β1 receptors and germ cell-secreted fibronectin. The cellular motion itself is, unexpectedly, independent of these integrin receptors.

Bone development, as a physiological process, has no parallels in its enmeshment with ECM dynamics. With dual live-cell imaging of collagen and osteocyte dynamics in the murine model, Shiflett et al. gain new insights into the dynamic ECM assembly processes and osteocyte entrapment in the collagen matrix that characterize bone development and remodeling. Their results suggest multiple mechanisms for osteocyte entrapment in the collagen matrix that are largely directed by ECM dynamics.

Epithelial-Mesenchymal Transition (EMT) is a fundamental characteristic that defines diverse developmental and pathological processes. Although the intracellular signaling and regulatory processes involved in EMT have received wide attention, an understanding of how tissue mechanics, and ECM mechanics in particular, affect this cell morphological transformation is lacking. The review by Scott et al. contributes to this poorly understood aspect of EMT by discussing evidence for the involvement of mechanochemical signaling of the ECM in EMT. Their work emphasizes the importance of bidirectional influences in which ECM mechanosignaling impinges on EMT and vice versa while highlighting the prominent roles played by non-structural matricellular proteins.

## Bioengineering

The importance of utilizing tissue engineering applications in the clinical setting with a clear understanding of the potential for ECM dynamics to shape/influence therapeutic outcomes is demonstrated by Wu et al.. Using human salivary stem/progenitor cells encapsulated in biocompatible hyaluronate-based hydrogels, they examine microstructure formation, growth, and reorganization with time-lapse imaging. Their results support an active role for the ECM components and integrin-mediated signaling in directing tissue assembly.

Advances in time-lapse imaging methods have proved critical for gaining a better understanding of ECM dynamics. These advances have been instrumental for the study of blood vessel formation and growth. Rauff et al. provide a thorough overview of the time-lapse imaging techniques that have helped reveal the multiple facets of ECM dynamics—a critical factor in shaping the interactions between a blood vessel and its microenvironment during sprouting angiogenesis.

Although great strides have been made in the development of methods for measuring and evaluating the biomechanics of ECM assembly/maintenance in various tissues and cell assemblies, studies investigating the local material and mechanical properties of matrix in an *in vivo* 3D tissue are scarce. Akos et al. attempt to alleviate this deficiency by taking the initial steps in measuring the local viscoelastic properties of ECM during avian embryogenesis. The authors devise a magnetic force-based rheometry method to measure the local rheological properties of the mesoderm-endoderm interface within quail embryos. By tracing the rotational displacement of injected ferromagnetic nanorod probes, they measure the local elasticity of the embryonic tissue at a given region of interest. More investigations along these lines are expected in future studies to help render a “viscoelasticity map” for the whole embryo. Extending this line of investigation further, it is now possible to imagine the construction of maps depicting the fluctuating material properties as cells and ECM determine/direct the forces that shape the embryo.

## Pathology

Tucker and Degen's review of tenascin-W—a member of the family of chordate ECM—describes its discovery, architecture, evolution, and expression patterns while highlighting its role in osteogenesis and adult stem cell niches. The authors also discuss the translational research merits of considering tenascin-W as a potential target for anti-cancer therapies due to its prominence in many solid tumors and its absence in the ECM of most adult tissues.

Pattar et al. review the clinical potential of ECM bioscaffolds to attenuate cardiac remodeling, and to restore ECM homeostasis by their ability to steer endogenous mechanisms of tissue repair following post-myocardial infarction ischemic injury. The array of ECM bioscaffolding options discussed in this review reveals a window of opportunity available for use as an adjunct therapy to surgical revascularization. An ideal ECM bioscaffold could positively alter the course of fibrotic remodeling, which often complicates the recovery of cardiac pump function.

Cleft lip/palate is among the frequently occurring congenital anomalies. ECM dynamics is a critical, yet poorly understood, component of palatogenesis. The review by Paiva et al. offers the current perspectives on both palatogenesis and on the occurrence of cleft lip/palate in terms of ECM dynamics and its dysregulation.

In their presentation of epithelial ECM dynamics under pathological contexts, Bhattacharjee et al. discuss the myriad dimensions of ECM-immune cell signaling exemplified during skin diseases. The effects on the immunomodulatory roles of cells and on ECM dynamics by atopic dermatitis, psoriasis, epidermolysis bullosa, and skin cancers are their primary focus.

## Summary

Each article included in this collection is a vignette that captures the intricacies and idiosyncrasies of ECM dynamics in diverse contexts within the realms of biology, bioengineering, and pathology. We hope that this collection will both inform and inspire the students of ECM dynamics working across various disciplines.

## Author Contributions

All authors listed have made a substantial, direct and intellectual contribution to the work, and approved it for publication.

## Conflict of Interest

The authors declare that the research was conducted in the absence of any commercial or financial relationships that could be construed as a potential conflict of interest.

